# Increased sub-clinical levels of autistic traits are associated with reduced multisensory integration of audiovisual speech

**DOI:** 10.1038/s41598-019-46084-0

**Published:** 2019-07-02

**Authors:** Thijs van Laarhoven, Jeroen J. Stekelenburg, Jean Vroomen

**Affiliations:** Department of Cognitive Neuropsychology, Tilburg University, P.O. Box 90153, 5000 LE Tilburg, The Netherlands

**Keywords:** Human behaviour, Perception

## Abstract

Recent studies suggest that sub-clinical levels of autistic symptoms may be related to reduced processing of artificial audiovisual stimuli. It is unclear whether these findings extent to more natural stimuli such as audiovisual speech. The current study examined the relationship between autistic traits measured by the Autism spectrum Quotient and audiovisual speech processing in a large non-clinical population using a battery of experimental tasks assessing audiovisual perceptual binding, visual enhancement of speech embedded in noise and audiovisual temporal processing. Several associations were found between autistic traits and audiovisual speech processing. Increased autistic-like imagination was related to reduced perceptual binding measured by the McGurk illusion. Increased overall autistic symptomatology was associated with reduced visual enhancement of speech intelligibility in noise. Participants reporting increased levels of rigid and restricted behaviour were more likely to bind audiovisual speech stimuli over longer temporal intervals, while an increased tendency to focus on local aspects of sensory inputs was related to a more narrow temporal binding window. These findings demonstrate that increased levels of autistic traits may be related to alterations in audiovisual speech processing, and are consistent with the notion of a spectrum of autistic traits that extends to the general population.

## Introduction

Autism Spectrum Disorder (ASD) is a pervasive neurodevelopmental disorder characterized by restricted interests, repetitive behaviour and deficits in social communication^1,2^. Although widely reported in ASD^3^, atypical sensory processing was only recently included as a core diagnostic criteria for ASD with the introduction of the DSM-5^2^. Emerging evidence suggests that many of the atypical sensory experiences seen in ASD may stem from a general inability to properly integrate sensory information from multiple modalities into accurate and meaningful percepts^4–6^.

Evidence supporting this notion has been widely – but not exclusively^7–9^ – reported in studies examining audiovisual speech perception in ASD^10^. In typically developing (TD) individuals, integration of multimodal inputs allows the brain to process sensory information more efficiently and provides significant perceptual benefits. Lip-reading under noisy listening conditions, for instance, significantly improves speech intelligibility^11,12^. Compared to TD controls, individuals with ASD tend to benefit less from visual articulatory cues when listening to noise-masked speech, indicating that they show alterations in multisensory integration (MSI) of audiovisual speech^13–16^. While visual cues are especially useful under suboptimal listening conditions where the auditory signal is degraded, visual input may also affect auditory perception of clearly audible speech. A prime example of this is the McGurk illusion^17^, in which the presentation of an incongruent audiovisual stimulus pairing (e.g. auditory /ba/ visual /ga/) typically induces an illusory percept (e.g. /da/). Previous research shows that individuals with ASD are less susceptible to the McGurk illusion compared to TD controls^18–23^. This reduced perceptual binding suggests that speech perception in ASD is less affected by visual input, and hence more biased towards the auditory modality. A possible underlying cause of these alterations in MSI is that individuals with ASD have impaired temporal binding abilities for multisensory speech signals^24^. To benefit from lip-read cues in audiovisual speech perception, one must be able to assess whether the incoming auditory and visual information should be integrated into a unified percept. One of the most important cues indicating that multisensory input should be bound together is temporal proximity^25^. Being able to perceive the relative timing of incoming sensory signals from multiple modalities is thus vital to properly integrate audiovisual speech. Several studies have shown that individuals with ASD have reduced temporal acuity and a wider temporal binding window (TBW) for speech stimuli compared to TD controls^26–28^. Evidence for an explicit link between multisensory temporal processing and audiovisual perceptual binding is found in TD^29,30^ and ASD populations^24,31^, suggesting that the atypical patterns of MSI observed in ASD might indeed be linked to alterations in multisensory temporal processing.

The current study aims to investigate whether autistic traits in the general population are related to MSI. As a spectrum disorder, symptoms of ASD are found to varying degrees in the general population^32^. Given the presumed relationship between MSI and ASD in clinical populations, one might expect that, in the general population, MSI and sub-clinical autistic symptoms are associated as well. However, it is unclear whether there is a steady decrease of MSI with increasing severity of ASD (across subclinical and clinical groups), or if atypical patterns of MSI may only emerge when a certain (clinical) threshold of severity of ASD is exceeded. To our knowledge, only four studies have examined the impact of sub-clinical levels of autistic traits on MSI^33–36^. One study examined the relationship between autistic traits and susceptibility to the McGurk illusion in a Japanese sample of 46 TD individuals^35^. Autistic traits were assessed via the Adult Autism Spectrum Quotient (AQ) self-report questionnaire. The AQ is a widely used screening instrument for ASD that assesses five subdomains associated with autistic symptomatology: *social skill*, *attention switching*, *attention to detail*, *communication* and *imagination*^37,38^. The experiment included auditory, audiovisual congruent and audiovisual incongruent stimulus presentations of the utterances /pa/, /ta/ and /ka/. The results showed that in the incongruent condition, AQ score was *negatively* correlated with the rate of fused (McGurk) responses (e.g. /ta/ in response to auditory /pa/ visual /ka/), but positively correlated with auditory responses (e.g. /pa/ in response to auditory /pa/ visual /ka/). This suggests that – similar to clinical ASD populations – speech perception in TD individuals with higher AQ scores is less affected by visual input, and more reliant on the auditory modality. However, another study using a similar experimental design but with the addition of background noise found that AQ score was *positively* correlated with fused responses for McGurk stimuli^36^. These inconsistencies have not been addressed to date, so it is unclear if these mixed findings are caused by differences in participant populations or experiment-related factors.

Another study examined the relationship between multisensory temporal processing and autistic traits using a simultaneity judgement (SJ) task wherein 101 TD participants reported whether an auditory beep and a visual flash occurred at the same time or not^33^. The results showed that the point of subjective simultaneity (PSS) – the stimulus onset asynchrony (SOA) at which a participant most likely perceived the auditory and visual stimuli as occurring simultaneously – was related to autistic traits assessed via the AQ, with the PSS shifting toward an auditory-leading bias as autistic symptoms increased. More specifically, individuals with higher AQ scores and increased difficulties in the ability to switch attention had a stronger tendency to report auditory-leading stimulus presentations as occurring simultaneously. One interpretation of this shift toward an ecologically less valid point is that individuals in the general population with higher levels of autistic traits prioritize auditory information over visual information; which is in line with the presumed over-reliance on the auditory modality observed in ASD^19,20,23^. Another explanation for this finding is that individuals with more ASD traits have a decreased ability to infer the probabilistic structure of sensory events. Without a precise internal probabilistic representation of the environment, their perception may be less affected by prior experience and more driven by sensory input^39–42^. Evidence for this interpretation is found in another study that examined how multisensory temporal adaptation is related to sub-clinical symptoms of ASD measured by the AQ^34^. Using a statistical learning paradigm including visual flashes and beeps, 60 TD participants were exposed to three-minute adaptation sessions of synchronous, auditory-leading and visual-leading audiovisual stimulus presentations. After exposure to visual-leading stimulus pairings, the participants’ perception of synchrony shifted towards visual-leading presentations, as was reported before in TD^43^. The strength of this temporal recalibration effect was significantly related to the level of autistic traits that participants exhibited. Specifically, an increased tendency to focus on local details of sensory input was related to weaker temporal recalibration. This suggests that individuals with increased levels of autistic traits are indeed less able to utilize temporal regularities in the environment, and that their perception may thus be less affected by prior expectations and more driven by sensory input.

Taken together, the results of these studies^33–36^ suggest that sub-clinical levels of autistic traits are indeed related to alterations in MSI. However, the studies to date that examined the impact of autistic traits on audiovisual temporal processing only used artificial stimuli (i.e. beeps and flashes)^33,34^, so it is unclear whether the results of these studies extent to more natural stimuli with higher ecological validity such as audiovisual speech. The studies that did use (elementary components) of speech to examine the relationship between autistic traits and MSI yielded inconsistent results^35,36^, and it remains to be elucidated whether these mixed results are caused by differences in participant populations or experiment-related factors. Furthermore, AQ subdomain scores were not reported in these studies^35,36^, so it is unclear how autistic traits in the general population within each subdomain of autistic symptomatology relate to MSI of audiovisual speech.

Here, we examined the relationship between sub-clinical levels of autistic traits and MSI of audiovisual speech in a large population of TD individuals (*N* = 101). ASD traits were assessed via the AQ^37^. The primary measures of MSI were audiovisual perceptual binding (assessed with a McGurk task), visual enhancement of speech intelligibility (assessed with a speech-in-noise task) and audiovisual temporal processing (assessed with an SJ task). To our knowledge, this is the first study to link sub-clinical autistic traits to MSI of audiovisual speech using several assessments in a within-subjects design. This approach allowed for a direct comparison of results across paradigms and aimed to further determine the specificity of the relation between autistic symptomatology and MSI in the general population.

## Results

### Autism spectrum quotient

Descriptive statistics of the average total AQ and subscale scores are presented in Table 1. Total AQ score ranged from 8 to 32 with a mean of 17.33 (*SD* = 4.88), which is in line with the expected average total AQ score of a non-clinical population^32^.

**Table 1:** Descriptive statistics of the average total AQ and subscale scores.

	Mean (SD)	Range
Total AQ (0–50)	17.33 (4.88)	8–32
Social skill (0–10)	2.30 (2.03)	0–9
Imagination (0–10)	2.04 (1.64)	0–7
Attention to detail (0–10)	5.38 (2.29)	1–10
Attention switching (0–10)	4.96 (1.89)	1–9
Communication (0–10)	2.65 (1.92)	0–8

*AQ* Autism Spectrum Quotient.

### McGurk task

**Figure 1:**
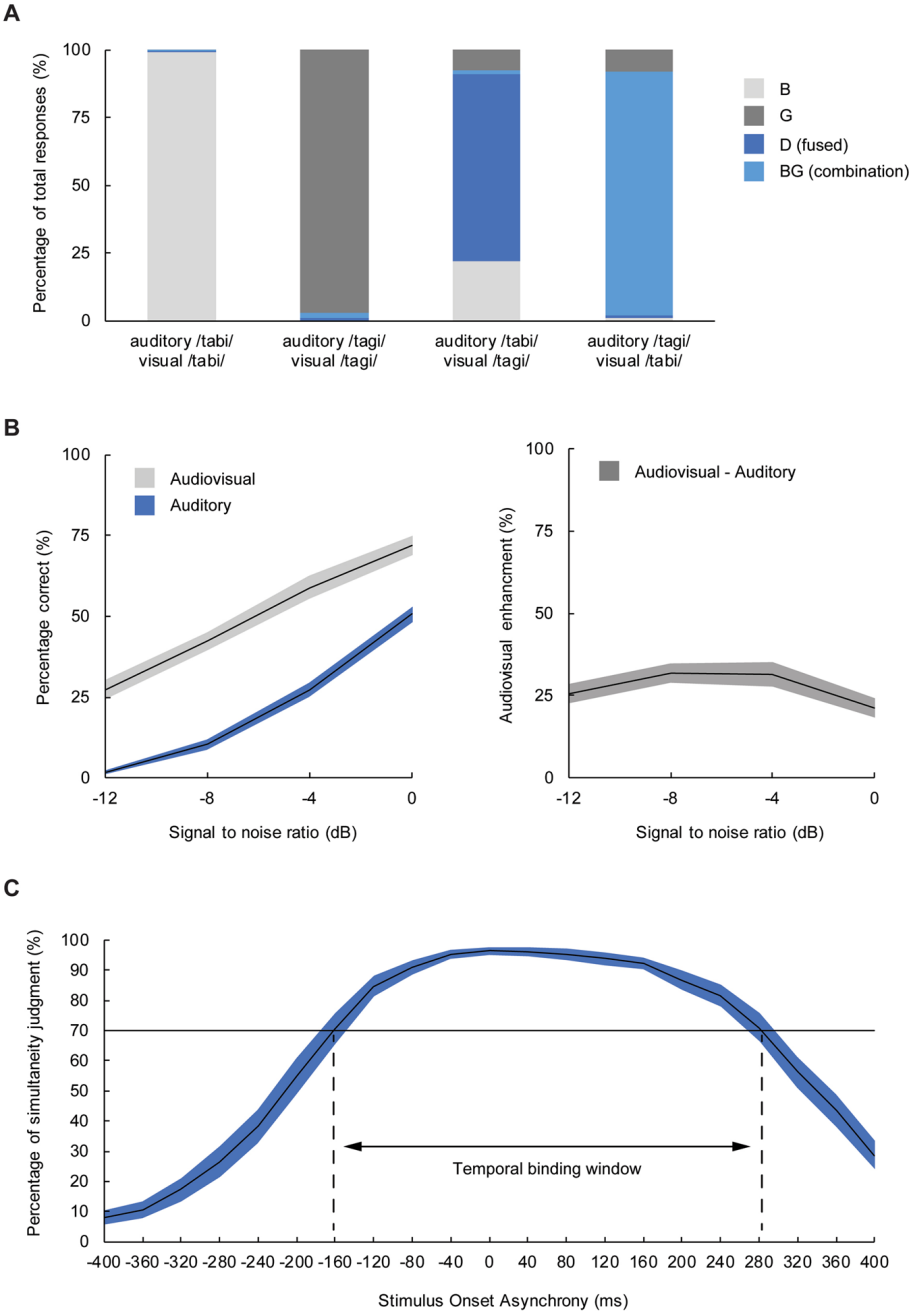
Overview of the behavioural data from each experimental task. Panel A: Grand average response rates for each audiovisual stimulus pairing presented in the McGurk task. Trials included audiovisual congruent (auditory /tabi/ visual /tabi/; auditory /tagi/ visual /tagi/) and audiovisual incongruent (auditory /tabi/ visual /tagi/ [fused]; auditory /tagi/ visual /tabi/ [combination]) stimulus pairings. Possible responses to the stimuli were auditory (B or G), visual (B or G), fused (D) or combination (BG). Panel B: Grand average word recognition performance for each condition (auditory, audiovisual) included in the speech-in-noise task and audiovisual enhancement (audiovisual – auditory performance) as a function of signal-to-noise ratio (SNR). Shaded areas represent 95% confidence intervals. Panel **C**: Grand average percentages of simultaneity judgment (SJ) for each stimulus onset asynchrony (SOA) included in the SJ task. Shaded area represents the 95% confidence interval. For each participant, percentages perceived as synchronous were calculated for each SOA. Two separate logistic curves were fitted on the negative (auditory-leading) and positive (visual-leading) SOAs, respectively. The TBW was calculated for each participant as the difference in ms between the SOAs at which the y-value of the logistic curves equalled 70%^25^.

Mean response rates were calculated for each of the four conditions (Fig. 1, panel A). Mean percentages auditory responses were submitted to a repeated measures MANOVA with the within subjects factor Stimulus (auditory /tabi/ visual /tabi/; auditory /tagi/ visual /tagi/; auditory /tabi/ visual /tagi/; auditory /tagi/ visual /tabi/). Average percentages auditory responses to the congruent stimulus pairings were high (99% and 97% for auditory /tabi/ visual /tabi/ and auditory /tagi/ visual /tagi/, respectively), indicating that participants were able to correctly identify the syllables for natural stimulus pairings. The MANOVA revealed a main effect of Stimulus *F*(3, 98) = 1077.64, *p* < 0.001, *η*_p_^2^ = 0.97. Post hoc paired samples t-tests (Bonferroni corrected) showed that there was no difference in correct responses between the two congruent stimulus pairings, and that the average percentage auditory responses was lower for incongruent than congruent stimulus pairings (all *p* values < 0.001) – indicating the occurrence of the McGurk illusion. Furthermore, the average percentage of auditory responses to the incongruent stimulus pairing auditory /tabi/ visual /tagi/ was higher (22%) than the average percentage of auditory responses to the incongruent stimulus pairing auditory /tagi/ visual /tabi/ (8%); *t*(100) = 4.34, *p* < 0.001, *d* = 0.53).

To examine the associations between autistic traits and perceptual binding, the percentages of fused and combination responses to the incongruent stimulus pairings (i.e. /tadi/ responses to the stimulus pairing auditory/tabi/ visual /tagi/, and /tabgi/ responses to the stimulus pairing auditory /tagi/ visual /tabi/) were calculated for each participant. In addition, individual percentages of auditory responses to each incongruent stimulus pairing were calculated to examine a potential bias towards the auditory modality. These indices of perceptual binding were then correlated with individual total AQ and subscale scores, and indices of visual enhancement of speech-in-noise and temporal processing (see below).

### Speech-in-noise task

Responses were checked for typographical errors and scored as either correct or incorrect. For each participant, percentages of correctly recognized words were calculated for each signal-to-noise ratio (SNR) in both the audiovisual (AV) and auditory (A) condition. Grand average percentages of correct responses for each condition as a function of SNR are shown in Fig. 1 panel B. A two-way repeated measures MANOVA including the within subjects factors condition (AV, A) and SNR (0, −4, −8, −12 dB) revealed a two-way interaction between these factors *F*(3, 98) = 11.22, *p* < 0.001, *η*_p_^2^ = 0.26. Post hoc paired samples t-tests (Bonferroni corrected) showed that the average percentage correctly identified words was on average 28% (*SD* = 10.28) higher in the AV condition compared to the A condition at all SNRs (all *p* values < 0.001), thereby replicating numerous studies showing that observing a speaker’s articulatory movements can substantially enhance speech comprehension under suboptimal listening conditions^11,12,44^.

In accordance with previous research on audiovisual speech perception in noise in ASD^14^, visual enhancement of speech intelligibility (AV gain) was indexed for each participant as the difference in percentage correctly recognized words between the AV and A condition (AV–A) averaged across all four SNRs.

### Simultaneity judgment task

For each participant, percentages perceived as synchronous were calculated for each SOA. Two separate logistic curves were fitted on the negative (auditory-leading) and positive (visual-leading) SOAs, respectively. The TBW was calculated for each participant as the difference in ms between the SOAs at which the y-value of the logistic curves equalled 70%^25^. Data from 14 participants were excluded from the analyses because their calculated temporal binding window exceeded the boundaries of the SOAs included in the task, indicating that they did not adhere to the task instructions or were unable to perform the task correctly. Simultaneity judgment percentages for each SOA averaged across the remaining 87 participants are shown in Fig. 1 panel C. The average TBW width was 502.35 ms (*SD* = 138.98), which is similar to previous research on the TBW for audiovisual speech^30^.

### Correlation effects

Pearson product-moment correlation coefficients (bivariate) were calculated to determine the relationships between the total AQ and subscale scores, AV gain and TBW. Spearman’s rank-order correlation coefficients were computed to examine the relationships between the AQ and subscale scores, AV gain, TBW, and perceptual binding indices since the average percentages of reported fused, combination and auditory responses in the McGurk task were not normally distributed. The multiple comparisons problem was addressed with the Benjamini-Hochberg procedure^45^ with a false discovery rate of 0.05. Non-significant correlations were further examined with Bayesian correlation tests using a default prior width of 1 (JASP version 0.9.2, https://jasp-stats.org/) to determine if the data support the null hypothesis over the alternative hypothesis. A Bayes Factor (BF_01_) larger than 1 indicates that the data support the null hypothesis, while a BF_01_ smaller than 1 indicates that the data support the alternative hypothesis. Data were interpreted as anecdotal, moderate, or strong evidence in favour of the null hypothesis if the BF_01_ was between 1–3, 3–10, and 10–30, respectively^46^.

### Autistic traits

We first examined the correlations between the different AQ subscales. There was a significant relationship between the subscales social skill and attention switching (*r* = 0.31, *p* = 0.002), and between the subscales social skill and communication (*r* = 0.31, *p* = 0.002). Since the subscales social skill and communication were not significantly related to any of the measures of MSI, these correlations will not be further discussed here. Correlations between the subscales imagination, attention switching and attention to detail were all non-significant (all *p* values > 0.39). Further examination of these non-significant correlations using Bayesian correlation tests provided moderate evidence for the null hypothesis (all BF_01_ between 5.60 and 7.93), indicating that these subscales likely assessed different subdomains of autistic symptomatology.

### Correlations between measures of MSI

There was a negative correlation between audiovisual enhancement and percentage of auditory responses to the incongruent McGurk stimulus pairing auditory /tabi/ visual /tagi/ (*r*_*s*_ = −0.22, *p* = 0.03), but this relationship did not remain significant after adjustment for multiple comparisons using the FDR controlling procedure. A Bayesian correlation test provided only anecdotal evidence for the alternative hypothesis (BF_01_ = 0.67), which suggests that, although the current results could be indicative of a relationship between audiovisual enhancement and auditory responses to incongruent McGurk stimuli, the current data are insensitive to detect a correlation between these indices. There were no other significant correlations between the indices of perceptual binding, audiovisual enhancement, and audiovisual temporal processing (all *p* values > 0.05). Bayesian correlation tests provided anecdotal evidence for the null hypothesis for correlations between the indices of perceptual binding (other than auditory responses to incongruent McGurk stimuli, see above) and audiovisual enhancement (all BF_01_ between 1.39 and 3.06.), indicating that the current data were insensitive and therefore unable to provide support for the lack of a relationship between these indices. Bayesian correlation tests did provide moderate evidence for the null hypothesis for correlations between audiovisual temporal processing and audiovisual enhancement, and audiovisual temporal processing and perceptual binding (all BF_01_ between 3.07 and 7.25) – indicating that the temporal processing paradigm likely tapped into different processes of MSI than the paradigms used to assess audiovisual enhancement and perceptual binding, and, importantly, that significant associations between autistic traits and these measures of MSI were likely not interdependent.

### McGurk task

**Figure 2:**
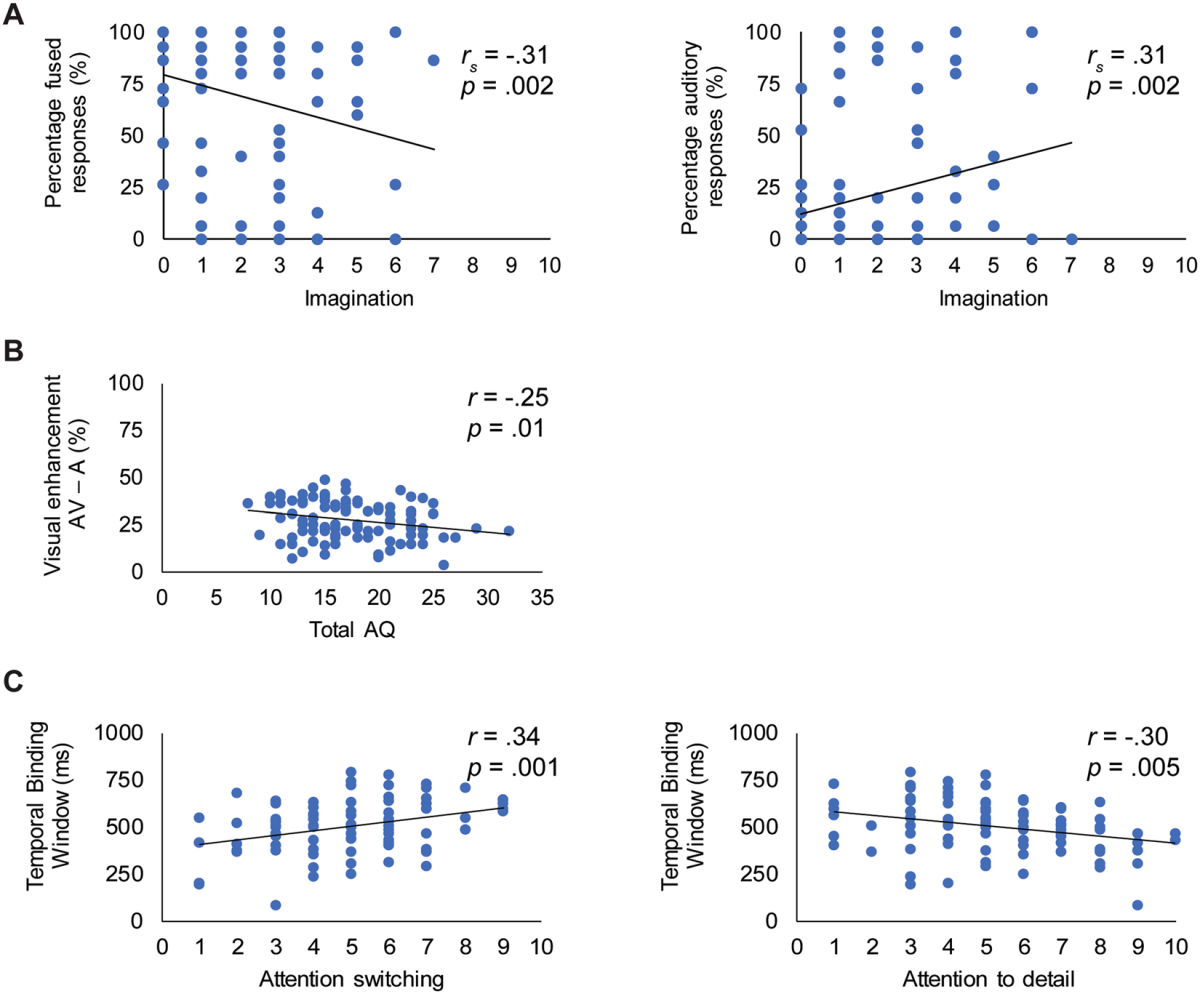
Several significant correlations were found between specific subdomains of autistic traits and audiovisual speech processing. Panel A: The subscale *imagination* was significantly related to audiovisual perceptual binding of incongruent McGurk stimuli. Individuals reporting higher (more autistic-like) scores on the subscale imagination reported fewer fused responses, but more auditory responses to the incongruent stimulus pairing auditory /tabi/ visual /tagi/ compared to individuals with lower scores on this subscale. Panel B: Increased overall autistic-like behaviour (indexed by *total AQ* score) was associated with reduced visual enhancement of speech intelligibility in noise (indexed by audiovisual (AV) – auditory (A) performance). Panel C: Participants experiencing increased difficulties with *attention switching* (and exhibiting more rigid and restricted behaviour) were more likely to bind audiovisual speech stimuli over longer temporal intervals, while increased *attention to detail* (i.e. the tendency to focus on local aspects of sensory inputs) was related to a more narrow temporal binding window.

There was no significant correlation between total AQ and indices of perceptual binding assessed by the McGurk task (all *p* values > 0.05, all BF_01_ between 6.53 and 7.93). However, the subscale *imagination* was significantly related to audiovisual perceptual binding of incongruent McGurk stimuli. Individuals reporting higher (more autistic-like) scores on the subscale imagination reported fewer fused responses (*r*_*s*_ = −0.31, *p* = 0.002), but more auditory responses (*r*_*s*_ = 0.31, *p* = 0.002) to the incongruent stimulus pairing auditory /tabi/ visual /tagi/ compared to individuals with lower scores on this subscale (Fig. 2, panel A). Further examination revealed a negative correlation between the subscale imagination and percentage of combination responses to the incongruent stimulus pairing auditory /tagi/ visual /tabi/ (*r*_*s*_ = −0.20, *p* = 0.04), but this relationship did not remain significant after adjustment for multiple comparisons using the FDR controlling procedure. Bayesian correlation tests provided moderate evidence in favour of the null hypothesis (BF_01_ = 6.03), indicating that the relationship between these indices was indeed non-significant. There were no significant correlations between the other AQ subscales and indices of perceptual binding (all *p* values > 0.07, all BF_01_ between 5.46 and 8.03).

### Speech-in-noise task

*Total AQ* was significantly correlated with visual enhancement of speech embedded in noise (*r* = −0.25, *p* = 0.01). Participants with a higher total AQ showed less AV gain (i.e. AV–A) from lip-read information in the speech-in-noise task (Fig. 2, panel B). There were no significant correlations between any of the AQ subscales and AV-gain (all *p* values > 0.05, all BF_01_ between 1.56 and 6.93).

### Simultaneity judgment task

There was no significant correlation between total AQ and audiovisual temporal processing indexed by the TBW (*p* = 0.42, BF_01_ = 5.40). There was, however, a significant relationship between the subscale *attention switching* and TBW (*r* = 0.34, *p* = 0.001). Participants experiencing more difficulties with attention switching exhibited a wider TBW. The subscale *attention to detail* was negatively correlated with TBW (*r* = −0.30, *p* = 0.005). Participants with a stronger tendency to focus on small details of sensory input (at the expense of more coherent perceptions) exhibited a more narrow TBW (Fig. 2, panel C). There was a positive correlation between the subscale social skill and TBW (*r* = 0.24, *p* = 0.03), but this relationship did not remain significant after adjustment for multiple comparisons using the FDR controlling procedure. A Bayesian correlation test provided anecdotal evidence for the alternative hypothesis (BF_01_ = 0.66), which suggests that, although the current results could be indicative of a relationship between the subscale social skill and TBW, the current data are insensitive to detect a correlation between these indices. There were no significant correlations between the subscale imagination and TBW (*p* = 0.35, BF_01_ = 4.89), and between the subscale communication and TBW (*p* = 0.33, BF_01_ = 4.64).

## Discussion

Altered perception of audiovisual speech has been widely reported in ASD, including differences in perceptual binding and temporal processing, and impaired perception of noise-masked audiovisual speech^10^. To our knowledge, this study is the first to demonstrate that sub-clinical autistic traits are related to reduced audiovisual speech processing performance across multiple experimental paradigms assessing MSI. Associations between autistic traits and MSI were specific for the subscale *imagination* (reduced perceptual binding of incongruent McGurk stimuli), total *AQ* score (reduced audiovisual gain) and the subscales *attention switching* and *attention to detail* (wider and narrower TBW, respectively). There was no relationship between the subscales imagination, attention switching and attention to detail, or between any of the measures of MSI. The current results therefore demonstrate that autistic traits in TD individuals do not necessarily co-occur in every subdomain within the same individual, which is in line with the notion of a heterogeneous spectrum of ASD symptoms that extends to the general population. Importantly, the current results suggest that each subdomain of autistic traits may affect audiovisual speech processing abilities in a specific way.

### Perceptual binding and imagination

Reduced audiovisual perceptual binding − characterized by reduced fused responses to incongruent McGurk stimuli − has been widely reported in ASD^18–23^. Studies on the relationship between autistic traits and susceptibility to the McGurk illusion in the general population have yielded inconsistent results. Some have linked increased levels of autistic traits to reduced fused responses^35^, while others showed stronger fused responses for McGurk stimuli embedded in background noise^36^. The current study is in accord with previous work relating autistic traits to reduced audiovisual integration of incongruent McGurk stimuli^35^, and extends the existing literature by demonstrating that perceptual binding of incongruent audiovisual speech may be related to an individuals’ imagination abilities.

In the current study, individuals reporting a more limited (autistic-like) capacity to imagine reported fewer fused responses, but more auditory responses to the incongruent McGurk stimuli. This reduced perceptual binding behaviour is also found in clinical ASD populations^18–23^, and suggests that audiovisual speech perception in individuals with diminished (autistic-like) imagination abilities may be less affected by visual input, and more reliant on the auditory modality. Another explanation for the observed relationship between reduced susceptibility to the McGurk illusion and autistic-like imagination is that individuals with reduced imagination abilities may have a more literal perception of the world that is less affected by prior experiences, but more reliant on the sensory input^41^. Perceptual binding of incongruent audiovisual (i.e. McGurk) stimuli is primarily based on the prior expectation that auditory and visual stimuli that are presented in close spatial and temporal proximity are more likely to originate from the same external event, and should therefore be processed as a single unified percept^47–49^. Underweighting this prior expectation − ‘hypo-priors’, in Bayesian terms^41^ − could lead to a decreased tendency to automatically bind incongruent audiovisual speech inputs, which in turn may result in a more literal perception of the world in which individual components of audiovisual speech inputs are more likely to be perceived than the unified percept. Given that the auditory component of the McGurk stimuli in the current study was less ambiguous than the visual component, the hypo-priors account might be a plausible explanation of the relationship between autistic-like imagination and increased auditory responses to McGurk stimuli − at the expense of unified (i.e. fused) responses − found in the current study. Indirect evidence for this explanation is reported in a recent study examining recognition accuracy of low-pass filtered and thresholded grayscale images, so-called Mooney images^50^, in relation to autistic traits^51^. It was found that individuals with higher scores on the AQ subscale imagination were less likely to recognize Mooney images than those with lower scores, even after exposure to the original source images. This suggests that perception in individuals with more autistic-like imagination is indeed more literal and less susceptible to perceptual change. It should be noted that in one study, autistic traits were linked to *increased* perceptual binding of McGurk stimuli embedded in background noise^36^. Further research is therefore needed to examine the underlying mechanisms of the potential link between imagination abilities and perceptual flexibility, and the role of background noise. Still, the current results suggest that increased levels of autistic-like imagination may affect MSI of incongruent audiovisual speech.

### Visual enhancement of speech intelligibility in noise and ASD traits

Increased total AQ score was related to reduced visual enhancement of speech intelligibility in noise. Participants with increased levels of autistic-like traits showed less gain from lip-read information when perceiving noise-masked speech. Impaired audiovisual perception of noise-masked speech has been widely reported in ASD^13–16^. To our knowledge, this study is the first to report a relation between autistic traits and audiovisual speech-in-noise perception in a population of TD individuals, thereby demonstrating that alterations in audiovisual perception may be observed across a spectrum of ASD symptoms that extends to the general population. There was no specific link between audiovisual enhancement and any of the subdomains assessed by the AQ. This suggests that, in the current sample, individual differences in autistic traits within each subdomain may have been too subtle to impact audiovisual speech perception in noise, even though the cumulative impact of autistic traits across subdomains was significant.

### Audiovisual temporal processing, attention switching and attention to detail

The current results revealed a relationship between difficulties with attention switching and temporal processing suggesting that individuals with a stronger tendency to show rigid and restricted patterns of behaviour may bind audiovisual speech stimuli over longer temporal intervals. Analogous findings have been reported in a previous study showing that TD individuals with higher total AQ scores and increased difficulties with attention switching were more likely to perceive artificial audiovisual stimuli (i.e. beeps and flashes) as simultaneous when performing an SJ task than individuals with lower total AQ scores and less restricted patterns of behaviour, specifically for auditory-leading stimuli^33^. The current results are also in accordance with previous studies in clinical populations demonstrating wider TBWs for audiovisual speech stimuli in individuals with ASD^26–28^. Taken together, these findings suggest that individuals with increased levels of autistic traits associated with inflexible behaviour tend to have a *wider* TBW for audiovisual stimuli.

Autistic traits in the subdomain attention to detail were also related to temporal processing. Specifically, an increased tendency to focus on local aspects of sensory inputs (at the expense of global information) was associated with a more *narrow* TBW. This positive relationship between ASD traits and temporal precision may sound somewhat counter-intuitive, given that an *enlarged* TBW is generally assumed to reflect *decreased* temporal acuity^27^ – as it may result in the perceptual binding of stimuli that *should not* be bound together – while a narrow TBW, on the other hand, is assumed to reflect *increased* precision of multisensory temporal processing^52^. These seemingly contradictory results can be reconciled if we consider that *overly precise* temporal processing (i.e. temporal hyperacuity) may lead to the separate processing of stimuli that *should* be bound together. Evidence for this interpretation is reported in previous research on audiovisual temporal recalibration in TD individuals^34^, which demonstrated that the extent to which the visual-leading side of the TBW is malleable to temporal recalibration is related to the level of autistic symptoms exhibited in the attention to detail subdomain. Given that a certain degree of ‘tolerance’ to asynchronous (visual-leading) sensory input is required in temporal adaptation, defaulting towards a more narrow TBW may limit the range of temporal recalibration effects. Impairments in temporal recalibration have also been reported in clinical populations of individuals with ASD^53,54^, although it remains to be elucidated whether these impairments are specifically related to increased attention to detail.

The temporal binding window typically develops asymmetrically to reflect the statistics of the natural environment, where visual input typically arrives at the retina prior to auditory information reaches the cochlea. This results in a steeper slope of the left (auditory-leading) side compared to the right (visual-leading) side^47,55,56^. A TBW that is either too wide (diffuse) or too narrow (restricted) poorly reflects the temporal statistics of the environment, and may thus significantly impair an individuals’ ability to properly bind multisensory input. The current findings indicate that increased levels of autistic traits may be related to such alterations of the TBW. It has been hypothesized that these alterations of the TBW may have potential cascading effects on the perception of multisensory input such as audiovisual speech^52^. Evidence supporting this notion has been found in recent work in a clinical ASD population^31^, were a correlational pathway was found between multisensory temporal processing (indexed by the TBW) and audiovisual perceptual binding (indexed by the McGurk illusion). No such link was observed in the current study. In the aforementioned study^31^, no link between temporal processing and perceptual binding was found in a neurotypical control group. The participants included in the current study can be considered to be neurotypical, and, thus, the absence of a direct link between the indices of MSI in the current study is in line with previous work^24^. This shows that – as mentioned in the introduction – experimental observations in clinical ASD populations do not necessarily translate to individuals in the general population with subclinical autistic traits. For the speech in noise task it should be noted that the stimuli used in the SJ and speech-in-noise task in the current study were of different complexity (i.e. phonemes and nouns, respectively). The absence of a link between temporal processing and visual enhancement of speech intelligibility in noise could thus (in part) be explained by a difference between phoneme and whole-word perception. The lack of a direct link between visual enhancement of speech intelligibility in noise and susceptibility to the McGurk illusion is in line with a recent study showing no relationship between audiovisual sentence recognition in noise and susceptibility to the McGurk illusion^57^ – although the failure to find a direct link in the current study should not be considered as evidence that there is no relationship. Nevertheless, the current findings suggest that a cascading pathway of alterations in MSI from impaired temporal processing, through reduced perceptual binding, to impaired speech-in-noise perception is only found in clinical populations of individuals with ASD^24^. Hence, it could be speculated that the associations between the different subdomains of autistic traits and indices of MSI may be reliant on particular thresholds of overall autistic symptomatology. Further research is needed to unravel the various patterns of associations between autistic traits and MSI of audiovisual speech observed in the current study.

The current data suggest that potential subgroups characterized by a particular range of autistic-like behaviours and multisensory functioning may exist in the general population. An interesting avenue of research to pursue would therefore be to examine if similar subgroups can also be identified in clinical populations. Identifying potential subgroups may have important implications for conceptualisations of MSI in ASD. If specific alterations in MSI are indeed linked to distinct subdomains of autistic traits, the impact of these alterations might be reduced by explicit interventions. Previous research has demonstrated that in TD individuals, audiovisual speech-in-noise perception^58^ and temporal processing^59,60^ can be enhanced with training. However, the impact of training on audiovisual speech perception in ASD is still largely unknown. A recent study demonstrated that individuals with ASD exhibit typical rapid audiovisual temporal recalibration effects for phonemes^53^, which suggests that the TBW for audiovisual speech in ASD is malleable – although the longer-term effects are still unclear. Still, these findings suggest that audiovisual temporal acuity in ASD may be susceptible to perceptual training protocols. Another study showed that speech-in-noise performance in children with ASD may improve after extensive app-based audiovisual training^61^. However, the sample size of this study was very small (*N* = 4), and an untrained control group was not included, so further research is needed to corroborate these results. Still, MSI training in individuals with ASD seems to offer a promising avenue of research, that may ultimately reduce the impact of alterations in MSI on daily life of individuals with ASD.

### Study limitations

A limitation of the current study is that a visual-only condition was not included to control for potential individual differences in lip-reading abilities. It may therefore be questioned whether the observed associations between autistic traits and indices of MSI can partly be explained by higher or lower lip-reading abilities. To our knowledge, no study to date has related sub-clinical autistic traits to lip-reading performance. The literature on lip-reading in clinical ASD populations is inconclusive; while some studies have reported reduced lip-reading in ASD^14–16^, others have found that lip-reading is intact in ASD and comparable to neurotypical controls^18,21^. Still, this alternative account cannot be ruled out entirely. However, variability in lip-reading abilities likely would have had little effect on the observed links between temporal processing and autistic traits, since lip-reading is not essential for executing audiovisual simultaneity judgments. It is also unlikely that variability in lip-reading ability is solely responsible for the observed association between overall autistic symptomatology and audiovisual enhancement, as previous work has demonstrated that lip-reading abilities are not the driving factor in audiovisual enhancement of speech-in-noise perception in adolescents aged 13–15 years with and without ASD^14^. When extrapolating these findings to the current study, we may argue that for the even slightly older participants in the current study, lip-reading abilities are unlikely to explain differences in audiovisual enhancement indexed by the speech-in-noise task.

## Conclusions

The current study replicates previous findings demonstrating that autistic traits are found in varying degrees in the general population^32^. Importantly, this study reports a relationship between autistic traits and multiple indices of MSI of audiovisual speech in a non-clinical population. These findings demonstrate that increased autistic symptomatology may underlie alterations in audiovisual speech processing, not only in clinical populations of individuals with ASD, but also in TD individuals.

## Methods

### Participants

A total of 101 undergraduate students (86 female, mean age 20.10 years, *SD* = 2.45 from Tilburg University participated in this study. All participants reported normal hearing and normal or corrected-to-normal vision. None were diagnosed with a neurological disorder and none reported use of medication. All participants received course credits as part of a curricular requirement. Written informed consent was obtained from each participant prior to participation. The study was conducted in accordance with the Declaration of Helsinki. All experimental procedures were approved by the Ethics Review Board of the School of Social and Behavioral Sciences of Tilburg University (EC-2016.48).

### ASD trait assessment

Self-reported levels of autistic traits were assessed with the AQ^37^. The AQ is a widely used sensitive and reliable screening measure for autistic traits in the general population^37,62^, and comprises 50 statements examining five subdomains associated with ASD: *social skill* (“I find it easy to work out what someone is thinking or feeling just by looking at their face”), *attention switching* (“If there is an interruption, I can switch back to what I was doing very quickly”), *attention to detail* (“I often notice small sounds when others do not”), *communication* (“Other people frequently tell me that what I’ve said is impolite, even though I think it is polite”), and *imagination* (“If I try to imagine something, I find it very easy to create a picture in my mind”). Each scale is represented by 10 statements. Participants were instructed to read each statement very carefully and rate how strongly they agreed or disagreed with it on a 4-point Likert scale (definitely agree, slightly agree, slightly disagree, definitely disagree). Scores for each subscale can range from 0 to 10 and the total score on the questionnaire can range from 0 to 50, with higher scores indicating more symptoms of ASD. Poor social skill, poor communication skill, poor imagination, exceptional attention to detail and problems with attention switching (i.e. exhibiting more rigid and restricted patterns of behaviour) are associated with autistic-like behaviour. A total AQ score ≥ 32 is indicative of ASD^37^.

During a subsequent visit to the laboratory on a different day, participants completed a McGurk task, a speech-in-noise task, and an SJ task. Administering the AQ questionnaire and conducting the experimental procedures on two separate occasions ensured that participants were unaware of the fact that their AQ scores were correlated with their performance on the experimental tasks.

### Stimuli and experimental procedures

Participants were individually tested in a dimly lit and sound attenuated room and were seated in front of a 25-inch LCD monitor (BenQ Zowie XL2540) positioned at eye-level at a viewing distance of approximately 70 cm. Visual stimuli were presented on the 25-inch LCD monitor at a resolution of 1920 × 1080 pixels and a refresh rate of 240 Hz. Auditory stimuli were recorded at a sampling rate of 44.1 kHz and presented over two loudspeakers (JAMO S100) located directly to the left and the right of the monitor. Stimulus presentation was controlled using E-Prime 3.0 (Psychology Software Tools Inc., Sharpsburg, PA).

### McGurk task

Stimuli for the McGurk task were adapted from a previous study on perception of intersensory synchrony in audiovisual speech^63^, and consisted of audiovisual recordings of the pseudowords /tabi/ and /tagi/ pronounced by a male speaker. The entire face was visible on a neutral background and subtended approximately 9.80° horizontal (ear to ear) and 14.65° vertical (hairline to chin) visual angle. Videos were presented at a frame rate of 25 frames/s. Speech sounds were presented at a fixed level of 50 dB sound pressure level (SPL) at ear-level. Trials included audiovisual congruent (auditory /tabi/ visual /tabi/; auditory /tagi/ visual /tagi/) and audiovisual incongruent (auditory /tabi/ visual /tagi/ [fused]; auditory /tagi/ visual /tabi/ [combination]) stimulus pairings. All stimulus pairings were temporally synchronous and had a duration of 2000 ms. Each pairing was presented 15 times in random order (60 trials in total). Participants were instructed to carefully listen to the sounds and attentively watch the speakers lip movements on the monitor. After each trial participants reported what the speaker said by pressing one of four keys, “b,” “d,” “bg” or “g”. Task duration was approximately five minutes.

### Speech-in-noise task

Stimulus materials and experimental design were adapted from two previous studies on audiovisual speech perception^64,65^. Stimuli consisted of audiovisual recordings of 120 different simple mono- and disyllabic nouns pronounced by a male speaker (e.g., tree, room, sugar). The entire face of the speaker was visible on a neutral background and measured approximately 9.80° horizontally (ear to ear) and 14.65° vertically (hairline to chin). Videos were presented at a frame rate of 25 frames/s. Speech sounds were presented at a fixed level of 50 dB SPL at ear-level. Speech sounds were embedded in four levels of pink noise presented at 50, 54, 58 and 62 dB SPL, resulting in SNRs of 0, −4, −8 and −12 dB SPL. Noise onset was synchronized with video onset. The length of the videos (4 s) and auditory onset (1.5 s after video onset) were identical across all nouns.

Two conditions were included in the speech-in-noise task. In the audiovisual (AV) condition, nouns were presented in conjunction with the corresponding video of the speaker articulating the noun. In the auditory (A) condition, nouns were presented in conjunction with a still image of the speaker’s face (with closed mouth). To ensure that participants were less likely to anticipate the experimental condition prior to auditory onset, different still images were created for each noun by extracting still frames from the corresponding videos. A visual-only condition was not included since previous work using the same stimuli reported very low identification scores in unimodal lip-read word recognition^64^.

Eight of the 120 nouns included in the stimulus set were selected for a practice session that participants completed prior to the main experiment. The remaining 112 nouns were divided into eight subsets of equal size and difficulty. Subset difficulty was based on average viseme overlap^64^ and proportion of disyllabic versus monosyllabic nouns. Each condition (AV, A) × SNR (0, −4, −8, −12) combination was assigned to one of the eight subsets. The resulting 14 trials for each combination were presented in random order. To reduce possible item-specific effects, eight different stimulus lists were generated and counterbalanced across participants such that each condition × SNR combination was assigned equally to all subsets.

Participants were instructed to attentively listen to the speech sounds and watch the speaker’s face, and were informed that a real noun would be presented during each trial. After each trial, participants reported the word they heard using a computer keyboard. Participants were able to correct their answer. Pressing the return key confirmed an answer and initiated the next trial. Participants were allowed to take a short break after every 14^th^ trial to minimize potential fatigue effects. Total duration of the experiment was approximately 20 minutes. After the experiment, a list of all the nouns presented during the experiment was presented. To identify participants with insufficient vocabulary knowledge, participants were instructed to encircle the nouns they did not know the meaning of. Eight pseudowords were intermixed with the regular nouns in the list to control for the possible tendency of participants to report all words as known or not report unknown words as a social desirable response. The average percentage of unknown words was low (*M* = 3.42, *SD* = 3.79). No participants were excluded due to insufficient vocabulary knowledge.

### Simultaneity judgment task

Stimuli for the simultaneity judgment (SJ) task were adapted from a previous study on perception of intersensory synchrony in audiovisual speech^63^, and consisted of audiovisual recordings of the pseudoword /tabi/ pronounced by a male speaker. The entire face of the speaker was visible on a neutral background and measured approximately 9.80° horizontally (ear to ear) and 14.65° vertically (hairline to chin). SOAs were set relative to the visual onset moment of the speech. A total of 21 SOAs were included: −400, −360, −320, −280, −240, −200, −160, −120, −80, −40, 0, 40, 80, 120, 160, 200, 240, 280, 320, 360, 400 (all values in ms, negative values mean auditory-leading). Fifteen trials were presented for each SOA. The entire task included 315 randomly intermixed trials. After each trial, participants performed a two-alternative forced-choice task in which they indicated whether or not they perceived the presented sound and video as synchronous events. Total duration of the SJ-task was approximately 15 minutes.

## Data Availability

The datasets generated during and/or analysed during the current study are available from the corresponding author on reasonable request.
